# Mucin expression patterns in histological grades of colonic cancers in Ghanaian population

**DOI:** 10.11604/pamj.2017.27.267.9793

**Published:** 2017-08-10

**Authors:** Kwabena Owusu Danquah, Ernest Adjei, Solomon Quayson, Ernest Adankwah, Daniel Gyamfi, Paul Poku Sampene Ossei, Gideon Dzikunu, Portia Mensah, Cecilia Lepkor

**Affiliations:** 1 Department of Medical Laboratory Technology, Faculty of Allied Health Sciences, Kwame Nkrumah University of Science & Technology, Kumasi, Ghana; 2 Cancer and Infections Research Facility, College of Health Sciences, Kwame Nkrumah University of Science & Technology, Kumasi, Ghana; 3 Department of Pathology, School of Medical Sciences, College of Health Sciences, Kwame Nkrumah University of Science & Technology, Kumasi, Ghana; 4 Department of Pathology, Ghana Medical School, University of Ghana

**Keywords:** Mucins, histochemical staining, colorectal cancer, alcian blue

## Abstract

**Introduction:**

Myriad roles of mucins in normal tissues have been well documented, including lubrication of the epithelial surfaces; protection from physical damage; facilitation in cell-cell signaling and suppression of inflammatory activity. Pathological expression of mucins has been noted in cancer development and progression. This study sought to identify and quantify the types of mucins produced during various histological grades of colon cancer and to assess the diagnostic significance.

**Methods:**

Formalin fixed, paraffin-embedded tissue blocks, comprising three (3) normal colon and twenty-two (22) colon cancer tissues, were retrieved from the archives of the histopathology department of the Komfo Anokye Teaching Hospital. They were stained with Haematoxylin and eosin (H&E) for diagnosis and grading of tumours. Tissues were pre-digested with diastase and stained with Alcian blue (pH 2.5)/Periodic Acid Schiff to characterize the mucin variants present.

**Results:**

Our findings indicated that normal colonic tissues expressed exceptionally high amount of acid mucin and low amount of neutral mucin. However, there was a general decrease in mucin expression in colon cancers compared to normal colon tissues. Additional findings suggested that as cancer progresses from low grade to high grade of adenocarcinoma of the colon, there was generally a considerable decrease in the acid mucin production and an increase in the neutral mucin expression. In contrast, a sizeable subpopulation of high-grade adenocarcinomas of colon showed a rather opposite mucin expression pattern- increase in acid mucin and a decrease in neutral mucin.

**Conclusion:**

As colonic cancer progresses, there are corresponding changes in the mucin types and content such that there are decrease in acid mucin and increase in neutral mucin expressions.

## Introduction

Initiation and development of cancers, particularly colonic cancer is a very complex one with several variables playing crucial roles in sustaining the viability of the cancer. Even though initiations of colonic cancers are well attributed to genetic imbalances, which could be inherited [1] or somatic origin [2], other environmental under-linings have been proposed, including alcohol consumption [3, 4], tobacco use [5, 6], obesity [7, 8], radiations [9, 10] and physical inactivity [11]. It could be conjectured that some of latter variables target or influence genetic imbalances, but others are not well known or understood. It is well believed that the development of colonic cancers is influenced by several factors that are involved in molecular pathways leading to uncontrollable growth and metastasis of the cancer [12]. Development and progression of colonic cancers are well associated with abnormal expression of mucins. In the normal colon tissues, mucins function as a lubricant on surfaces to protect them from friction, erosion, harmful substances, unfavourable conditions and pathogens [13, 14]. Mucins of the normal colorectal tissues are predominately sulphated and carboxylated (acidic) mucins with scanty neutral mucins [15]. Sulphated mucosubstances have been found in the deeper mucosa of the colonic tissue, a location believed for cell divisions; this may suggest an important role of sulfated mucins in the control of cell division and that a decline in the production of the sulphated mucosubstances would predispose the colonic mucosa to malignancy [15]. Even though an increase production of mucin in adenocarcinomas of the colon has been reported some colonic cancers have been demonstrated to secret less mucins as compared to the normal [15]. No correlation has been established between the trend of secretion of the mucins and the degree of differentiation of the tumours. However, it is reported an overall reduction in the production of mucus in the colonic tissue while the proportion of neutral mucins was increased [16]. This study therefore aimed to identify mucins types and amount and correlate it with different grades of adenocarcinomas of colon.

## Methods

Twenty-five formalin fixed, paraffin embedded (FFPE) colonic tissues, which were retrieved from the archives of Histopathology Unit of the Department of Pathology, Komfo Anokye Teaching Hospital, Kumasi-Ghana, following ethical approval from both the hospital and Committee for Human Research, Publication and Ethics of School of Medical Sciences, KNUST. Two tissue sections at 3μm from each FFPE tissue block were produced. One batch of the sectioned FFPE colonic tissues was stained with Haematoxylin and Eosin (H&E) and were histologically diagnosed and graded by three independent pathologists. The second batch of the sectioned FFPE colonic tissues was dually stained with Alcian blue, pH 2.5 to demonstrate acidic mucins (sulfated and carboxylated) and Periodic Acid Schiff (PAS) to demonstrate glycogens and mucins. In order to exclusively demonstrate neutral mucins, dewaxed colonic sections were pretreated with diastase for 60 minutes to digest at existing glycogen in the tissue [17]. Positive and negative controls were run for quality assurance. The diastase-pretreated, Alcian Blue (pH 2.5)-Periodic Acid Schiff - AB (2.5)/DPAS technique, was used to differentiate acidic mucins and neutral mucins [18]. The amounts of mucins in the AB (pH 2.5)/D-PAS stained colonic sections were semi-quantified by 3 independent qualified biomedical scientists and graded as negative (-), low (+), mild (++), moderate (+++) and marked (++++) based on the intensity and the proportion of tissue/cells expressing mucins. Further, quantified acid and neutral mucins in each stained section was confirmed by measuring the intensity of deep blue (Alcian blue-acid mucins) and magenta (DPAS-neutral mucins) using Image J software, verse 1.48s (Rasband W, National Institutes of Health, USA) and Adobe Photoshop 2015.

## Results

**Figure 1:**
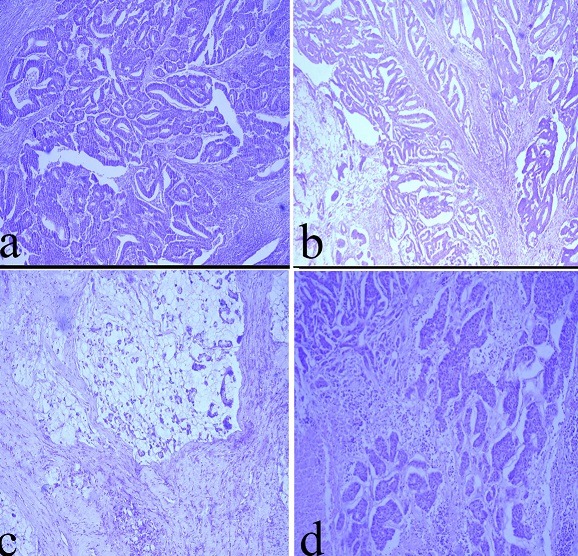
Photomicrographs of colon tissues stained with (H&E): (a) Grade 2, well-differentiated adenocarcinoma (b) Grade 2, moderate differentiated adenocarcinoma (c) Mucinous adenocarcinoma (d) Grade 3 poorly differentiated adenocarcinoma; all images scale = 10 μm

**Haematoxylin and eosin (H&E) based diagnosis and grading colonic cancer tissues:** Normal colon and colonic cancer tissues were H&E stained to confirm the diagnosis and grades of the tumours. Five of the 22 colonic cancers were diagnosed and graded as Grade 2, Well Differentiated Adenocarcinoma (G2WDA); 13 of the 22 colonic cancers as Grade 2, Moderately Differentiated Adenocarcinoma (G2MDA); 3 of the 22 colonic cancers as Grade 2 Adenocarcinomas (G2A) and only 1 case as Grade 3 Poorly Differentiated Adenocarcinoma (G3PDA) (Figure 1). The colonic cancers were broadly graded as low-grade adenocarcinomas (G2WDA) and high-grade adenocarcinomas (G2MDA, G2A, and G3PDA).

**Differential mucins staining with Alcian Blue/Diastase-Periodic Acid Schiff staining:** in order to correlate the mucin content/amount and types with different grading of colonic cancers, we performed Alcian blue (pH 2.5)/Diastase-Periodic Acid Schiff staining. The sensitivity and specificity of the staining mucin staining were tested using appropriate controls. This was also part of the quality assurance procedure. Quality controls for mucin demonstration For a positive control, normal colon stained blue for acid mucins using Alcian Blue (pH 2.5), Diastase Periodic Acid Schiff (DPAS) staining on prostate, and DPAS/Alcian blue (pH 2.5) staining on colon (results not shown). For a negative control, liver tissues stained negative for Alcian Blue (pH 2.5), indicating absence of acid mucins, but stained magenta in DPAS, indicating the presence of neutral mucins. Further, an omission of the periodic acid oxidation step in DPAS resulted in a negative staining on human liver (results not shown).

**Figure 2:**
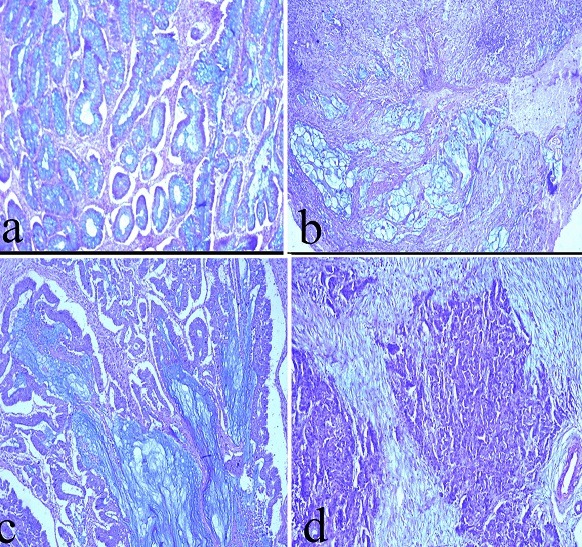
Photomicrographs of adenocarcinoma of the colon after staining with DPAS/Alcian Blue (pH 2.5): (a) Grade 2, well-differentiated adenocarcinoma (b) Grade 2, moderate differentiated adenocarcinoma (c) Mucinous adenocarcinoma (d) Grade 3 poorly differentiated adenocarcinoma; Alcian blue stains blue and D-PAS stains magenta; all images scale = 10 μm

**Mucin content/amount and types in colonic cancers:** in this study, the total mucin content in 22 colonic cancer cases was semi-quantified after double Alcian blue (pH 2.5) and Diastase-PAS staining. The mucin types (acid and neutral mucins) were also determined in each case. Both mucin content and types were estimated and correlated with tumour grade (Figure 2, Table 1). The average % acid mucin and neutral mucin contents of the 3 normal colon tissue was 36.7% and 1.9%, respectively (Table 1, Table 2), indicating that acid mucin is more predominate in human colon tissue than neutral mucin. The % acid mucin in the WDAs, which ranged from 21.2% to 41.9%, had an average % acid mucin of 41.9%. However, the % neutral mucin in the same tumour cases had a range from 4.0% to 10.4%, with an average % neutral mucin of 5.8%. Thirteen (13) colonic cancer cases which were dually stained with Alcian blue (pH 2.5) and D-PAS, showed some interesting findings. Unlike the normal colon and WDAs that showed a unique pattern of a predominately acid mucin, MDA showed a mixed pattern. Nine MDA cases showed an increase in acid mucin content against decrease neutral mucin content; 4 MDA cases revealed rather decrease acid mucin content with an increase neutral mucin content whereas 3 MDA cases showed no neutral mucin content (Figure 2, Table 1). The 3 cases of adenocarcinomas showed a mixed pattern similar to that of MDA (Table 2). In summary, the general trend of mucin expression in colonic cancer tissue indicated an increase in acid mucin and reduced neutral mucin contents.

**Table 1:** Expression patterns of mucins in colonic tissues

Histological Diagnosis	Grade	% AM	% NM	Intensity AM	Intensity NM
Normal colon	NA	43.6	3.9	+++	+
Normal colon	NA	45.5	1.8	+++	+
Normal colon	NA	21.0	0.1	++	-
WDA	2	21.2	10.4	++	+
WDA	2	48.0	5.4	+++	+
WDA	2	43.6	4.0	+++	+
WDA	2	46.6	5.2	+++	+
WDA	2	49.9	4.1	+++	+
MDA	2	11.4	27.2	+	++
MDA	2	42.4	6.1	++++	+
MDA	2	39.7	7.8	+++	+
MDA	2	24.7	11.0	+++	+
MDA	2	5.2	6.3	+	+
MDA	2	44.8	16.7	+++	+
MDA	2	3.9	5.6	+	+
MDA	2	41.3	2.1	++	+
MDA	2	19.7	0.0	++	-
MDA	2	22.6	54.9	++	+++
MDA	2	44.8	2.3	+++	+
MDA	2	32.9	0.0	+++	-
MDA	2	37.4	0.0	+++	-
MA	2	56.2	20.5	+++	+
Adenocarcinoma	2	7.5	19.7	+	++
Adenocarcinoma	2	28.3	33.2	+	++
PDA	3	36.0	4.2	++	+

AM: Acid mucin; NM: Neutral mucin; WDA: Well Differentiated Adenocarcinoma; MDA: Moderately Differentiated Adenocarcinoma; PDA: Poorly Differentiated Adenocarcinoma; MA: Mucinous Adenocarcinoma

**Table 2:** Relative mucin variant expression of the colonic tissues

Histological Diagnosis	Acid Mucin (%)	Neutral Mucin (%)	Ratio (AM/NM)
Normal Colon (3)	80.0	20.0	4
Low graded adenocarcinoma (5)	73.7	26.3	2.8
High graded adenocarcinoma (17)	63.7	36.3	1.8

AM is Acid mucin and NM is Neutral mucin

## Discussion

Mucins are well documented in playing essential physiological roles, including protecting epithelial surfaces against damages [13], suppressing inflammatory activity by preventing direct exposure of commensal bacteria to the epithelium [14] and the transmitting information from the external environment to the epithelium referred to as cell signaling [19]. In pathological conditions such as cancers, the functions of mucins have been highly noted elsewhere but limited information for those in African or Ghanaian population. This study therefore sought to assess mucins types and content in different grades of human colonic cancers using mucin histochemical analysis in Kumasi population. In order to achieve this, 25 colonic tissues consisting of 3 normal and 22 different graded colonic cancers, were histocemically stained to assess the types of mucins. Following histochemical staining (diastase-AB/PAS), it was observed that the normal colonic tissue demonstrated predominance of acidic mucin (80%) and scanty neutral mucin (20%), confirming what have been made already [15, 20] that acidic mucin is copiously present in the colon for the primary role of viscosity. A striking pattern, contrary to the amount of mucins in normal colon, was observed in low graded well-differentiated adenocarcinomas of the colon- a decrease in acid mucin (73.7%) and an increase in the neutral mucin expression (26.3%) as compared to the normal colon mucin variant expression (Table 2). Similarly, a further decrease in acid mucin (70.5%) and increase in neutral mucin production (29.5%) was observed in the moderately differentiated adenocarcinomas of the colon. The poorly differentiated adenocarcinoma and others designated as mucinous adenocarcinomas and adenocarcinomas of colon also respectively demonstrated much decrease in acid mucin production (66.7% and 53.8%) and a concomitant increase in neutral mucin production (33.3% and 46.2%). The findings from this study revealed that, there is a general decline in the acid mucin expression as the adenocarcinomas of the colon progressed from low-grade cancer such as well-differentiated adenocarcinoma through moderately differentiated adenocarcinoma to the high grade poorly differentiated adenocarcinoma whilst there was an corresponding upsurge in the expression of neutral mucins. This trend of increased expression of neutral mucin is consistent with the findings of Sugihara and Jass (1986) who indicated that qualitative changes occur in mucin expression in colorectal cancer cases and is associated with an increased neutral mucin production. Acid mucins have been suggested to inhibit tumour growth [21] as well as involved in the control of cell division-it is therefore tempting to suggest that a down regulation of acid mucin expression may then drive or predispose the colonic tissue to malignancy. Nonetheless, we also observed that a subgroup of moderately differentiated adenocarcinomas had rather an increase in acid mucins coupled with decrease neutral mucins. A similar finding has been reported in a chemically induced colonic cancer in rat, which showed a reduction in concentration of neutral mucins and sulfomucins and an increase in non-sulfated mucins [22]. Additionally, a similar pattern has been demonstrated in early gastric cancer [23]. Thus to the best of our knowledge, an increased acid mucins and reduced neutral mucin expression pattern has not demonstrated in colonic cancers in humans. Indeed, it will be of great importance to work on larger sample size of colonic cancer cases to determine whether an increased acid mucin-reduced neutral mucin expression is a predominate phenomenon among Ghanaian population, and also determine the biological significance of this finding in relation to prognosis and cancer aggressiveness.

## Conclusion

The pattern of mucin expression in the various grades of the colonic cancer generally indicated a remarkable decrease in acid mucin and an increase in neutral mucin expression as the cancers progress, however, a small subpopulation of the high-grade colonic cancers showed an opposing expression pattern. This novel finding does indicate the molecular heterogeneity of colonic cancers, which may also influence treatment regimens for colonic cancers.

### What is known about this topic

- Normal colorectal tissues produce or secrete copious amount of acidic (sulphated and carboxylated) mucins but scanty neutral mucins;
- It is widely postulated that sulfated mucins play a role in the control of cell division;
- Production of mucins, in general, is remarkably reduced in adenocarcinomas of colorectal tissues.

### What this study adds

- We, in this study, demonstrated that there is a progressive decline of acid mucin from low-graded to high graded adenocarcinoma of colorectal with corresponding increase in neutral mucins;
- We also demonstrated for the first time that a subgroup of moderately differentiated adenocarcinomas had rather an increase in acid mucins coupled with decrease neutral mucins in humans.

## Competing interests

The authors declare no competing interest.
